# Theta-Gamma-Modulated Synaptic Currents in Hippocampal Granule Cells In Vivo Define a Mechanism for Network Oscillations

**DOI:** 10.1016/j.neuron.2013.09.046

**Published:** 2014-01-08

**Authors:** Alejandro Javier Pernía-Andrade, Peter Jonas

**Affiliations:** 1 IST Austria (Institute of Science and Technology Austria), Am Campus 1, A-3400 Klosterneuburg, Austria

## Abstract

Theta-gamma network oscillations are thought to represent key reference signals for information processing in neuronal ensembles, but the underlying synaptic mechanisms remain unclear. To address this question, we performed whole-cell (WC) patch-clamp recordings from mature hippocampal granule cells (GCs) in vivo in the dentate gyrus of anesthetized and awake rats. GCs in vivo fired action potentials at low frequency, consistent with sparse coding in the dentate gyrus. GCs were exposed to barrages of fast AMPAR-mediated excitatory postsynaptic currents (EPSCs), primarily relayed from the entorhinal cortex, and inhibitory postsynaptic currents (IPSCs), presumably generated by local interneurons. EPSCs exhibited coherence with the field potential predominantly in the theta frequency band, whereas IPSCs showed coherence primarily in the gamma range. Action potentials in GCs were phase locked to network oscillations. Thus, theta-gamma-modulated synaptic currents may provide a framework for sparse temporal coding of information in the dentate gyrus.

## Highlights

• Granule cells in vivo fire action potentials sparsely but often in bursts
• Granule cells are exposed to barrages of fast excitatory postsynaptic currents
• Granule cells receive theta-coherent excitation but gamma-coherent inhibition

## Teaser

Pernía-Andrade and Jonas characterize the synaptic mechanisms underlying network oscillations and temporal coding in the dorsal hippocampus in awake rats. Theta-gamma-modulated synaptic currents may provide a framework for sparse temporal coding of information in the dentate gyrus.

## Introduction

**Figure 1:**
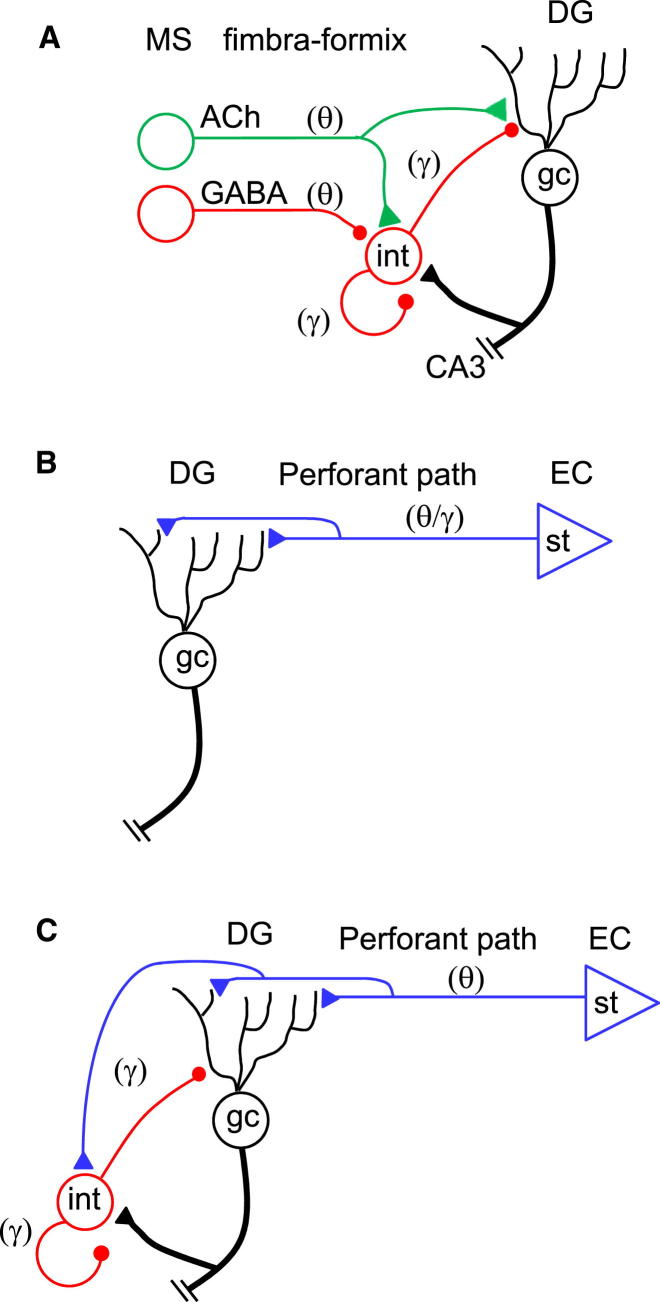
Circuit Models of Theta-Gamma Oscillations in the Dentate Gyrus (A) Classical model, in which theta rhythm originates in the medial septum and gamma rhythm is generated in interneuron loops. Adapted from Stewart and Fox (1990). For cholinergic innervation of the dentate gyrus, see Frotscher and Léránth (1986). (B) Alternative model in which both theta and gamma rhythms are relayed from the entorhinal cortex via glutamatergic synapses. Such a model is motivated by the finding that bilateral chronic lesions of the entorhinal cortex markedly reduce the power of both theta and gamma oscillations (Bragin et al., 1995). (C) Alternative model derived from the present experimental results. Theta rhythmic activity is primarily relayed from the entorhinal cortex via glutamatergic synapses, while gamma activity is generated intrinsically by local oscillators involving interneurons. Note that the schemes for simplicity only illustrate direct inputs to the dentate gyrus GC-interneuron network; polysynaptic pathways (e.g., septal and entorhinal input to entorhinal cortex neurons or hilar mossy cells) are omitted. MS, medial septum; DG, dentate gyrus; EC, entorhinal cortex; gc, granule cell; st, stellate cell; int, interneuron.

Network oscillations in the theta and gamma frequency range are thought to represent key reference signals for temporal encoding of information in neuronal ensembles (Buzsáki and Draguhn, 2004, Lisman and Jensen, 2013). The power of theta-gamma oscillations is particularly high in the dentate gyrus of the hippocampal formation (Bragin et al., 1995, Csicsvari et al., 2003). However, the underlying synaptic mechanisms are unclear (Buzsáki, 2002). The classical view suggests that theta activity is driven by cholinergic or GABAergic input from the medial septum (Stewart and Fox, 1990, Freund and Antal, 1988), while gamma activity is generated by GABAergic interneurons via recurrent or mutual inhibition mechanisms (Bartos et al., 2007; Figure 1A). In apparent contrast, previous studies demonstrated that theta-gamma oscillations in the dentate gyrus are markedly reduced by lesions of the entorhinal cortex (Bragin et al., 1995), suggesting a potential role of excitatory inputs for both theta and gamma rhythms in behaving animals (Figure 1B). However, the temporal structure of the excitatory input and its correlation with the local field potential (LFP) are unknown. Dissecting the synaptic mechanisms underlying rhythmic patterns in the LFP has remained difficult, since perisomatic inhibition and dendritic excitation produce indistinguishable current sink-source patterns (Mann et al., 2005).

Theta-gamma oscillations are thought to have important computational functions in the network. First, they may represent a reference signal for temporal encoding of information (Lisman and Jensen, 2013). Second, they facilitate communication between principal neurons by synchronization (Fries, 2009, Akam and Kullmann, 2010). Recent modeling suggested that gamma oscillations could also contribute to the selection of cells that receive the highest excitation level by a “winner takes all” mechanism (de Almeida et al., 2009a, de Almeida et al., 2009b). Such a mechanism may be particularly useful in the dentate gyrus, where it could potentially participate in both pattern separation and the conversion of grid into place codes (Hafting et al., 2005, Leutgeb et al., 2007). However, it is not known whether the properties of excitatory postsynaptic currents (EPSCs) and inhibitory postsynaptic currents (IPSCs) in hippocampal granule cells (GCs) are consistent with the predictions of such a model regarding temporal and spatial characteristics (e.g., gamma modulation and network coherence; de Almeida et al., 2009a, de Almeida et al., 2009b).

In the present paper, we intended to address three major questions. First, what is the firing pattern of mature hippocampal GCs in vivo in awake animals? This seemed critical, since recent work raised doubts regarding the identity of previously recorded cells in the dentate gyrus (Neunuebel and Knierim, 2012). Second, what is the temporal and spatial structure of the synaptic events underlying theta-gamma oscillations in the LFP? Third, does theta-gamma-modulated input contribute to coding and processing of information in the dentate gyrus? To address these questions, we used whole-cell (WC) patch-clamp recordings in vivo. GCs were rigorously identified by intracellular biocytin labeling, and synaptic activity was correlated with the simultaneously recorded LFP. We found that morphologically identified hippocampal GCs fired sparsely but preferentially in high-frequency bursts. Furthermore, synaptic currents were theta-gamma modulated, with theta-coherent excitation and gamma-coherent inhibition. Finally, action potentials were phase locked to nested theta-gamma oscillations. Thus, theta-gamma-modulated synaptic currents may provide a synaptic framework for temporal coding in the dentate gyrus (Lisman and Jensen, 2013). Part of the results was previously published in abstract form (A.J. Pernía-Andrade and P. Jonas, 2012, Soc. Neurosci., abstract).

## Results

### Sparse Action Potential Generation in Hippocampal GCs In Vivo

**Figure 2:**
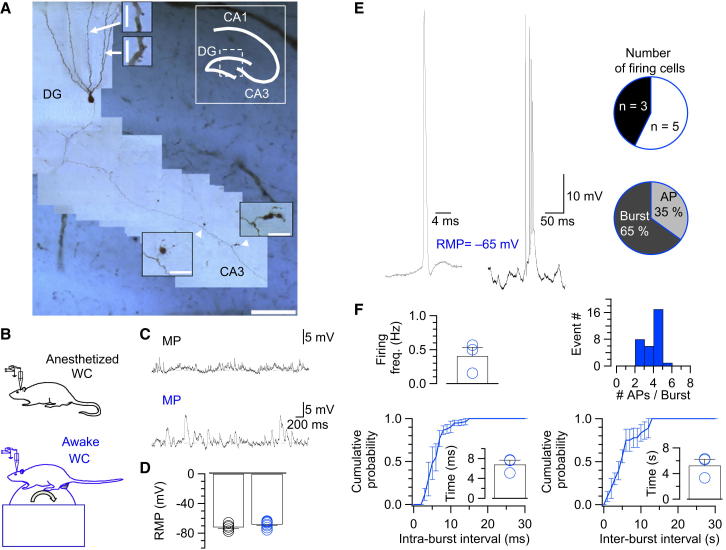
Sparse Action Potential Initiation and High Proportion of Bursts in Mature Hippocampal GCs In Vivo (A) Light micrograph of a GC filled with biocytin during WC recording and visualized by post hoc labeling with 3,3′-diaminobenzidine. Insets show spines on the GC dendrites (arrows) and large boutons emerging from the mossy fiber axon (arrowheads). Photomontage of images taken at different focal planes. Scale bars represent 10 and 100 μm for insets and main panel, respectively. Scheme illustrates hippocampal subfields, with rectangle depicting the region approximately corresponding to the photomicrograph. (B) Schematic illustration of the recording configurations (black, anesthetized; blue, awake rats). (C) Representative membrane potential recordings from dentate gyrus GCs in anesthetized (top) and awake (bottom) rats. (D) Summary bar graph of the resting membrane potential (RMP). Resting potential values were determined from temporal windows with minimal membrane potential variance. Black, anesthetized (five cells); blue, awake animals (eight cells). (E) Traces of “spontaneous” single action potential and action potential burst in awake rats. Pie charts illustrating the number of GCs firing ≥1 action potential during a 15–30 min recording period (top; black, firing cells; white, silent cells) and the proportion of single action potentials versus bursts in the subpopulation of firing GCs (bottom; dark gray, proportion of bursts; light gray, proportion of single action potentials). (F) Top left: summary bar graph of mean action potential frequency in the subpopulation of firing GCs. Top right: histogram of the number of action potentials per burst. Bottom: cumulative probability distributions and summary bar graphs of intraburst (left) and interburst (right) intervals. Bars represent mean ± SEM, circles indicate data from individual cells. Data in (E) and (F) were obtained from awake rats (eight cells). See also Figure S1.

The firing pattern of mature GCs in vivo is largely unclear (Neunuebel and Knierim, 2012). We therefore first determined the frequency of action potential initiation in rigorously identified mature GCs in vivo (Figure 2; Table 1). GCs in vivo showed periods of negative resting potentials (–71.9 ± 1.9 mV and –68.2 ± 1.5 mV in five anesthetized and eight awake rats, respectively) but also exhibited periods of depolarization and excessive membrane potential fluctuation (Figures 2C and 2D). In anesthetized rats, action potentials were absent in >15 min recording periods (five out of five cells; see Muñoz et al., 1990, Penttonen et al., 1997). In contrast, in awake rats, GCs generated spikes in three out of eight recordings (Figure 2E). However, all cells fired action potentials during depolarizing current injection, with maximal action potential frequency of 38 ± 1 Hz in anesthetized and 35 ± 3 Hz in awake rats (Figure S1 available online; Spruston and Johnston, 1992, Lübke et al., 1998). Thus, the absence of spikes was not due to a lack of intrinsic excitability under in vivo conditions. Surprisingly, in the subpopulation of firing GCs the proportion of single spikes was 35%, whereas the proportion of bursts was 65% ± 22%, with on average 3.3 ± 0.9 action potentials per burst (Figures 2E and 2F). Thus, GCs in vivo generated action potentials sparsely, but whenever they fired, preferentially fired in bursts.

**Table 1:** Properties of Hippocampal GCs In Vivo

	Anesthetized (mean ± SEM)	Awake (mean ± SEM)
Resting potential	–71.9 ± 1.9 mV (n = 5)	–68.2 ± 1.5 mV (n = 8)
Membrane potential standard deviation	1.2 ± 0.2 mV (n = 5)	1.9 ± 0.2 mV (n = 8)
Input resistance	151.0 ± 15.6 MΩ (n = 5)	143.0 ± 10.8 MΩ (n = 8)
Membrane time constant τ_m_	15.9 ± 3.2 ms (n = 5)	ND
Mean frequency of EPSCs^a^	15.7 ± 1.6 Hz (n = 15)	15.1 ± 1.6 Hz (n = 13)
Individual EPSC peak amplitude^b^	8.76 ± 0.69 pA (n = 15)	21.30 ± 2.4 pA (n = 13)
EPSC 20%–80% rise time	2.24 ± 0.06 ms (n = 15)	1.97 ± 0.06 ms (n = 13)
EPSC decay time constant^b^	5.95 ± 0.26 ms (n = 15)	3.84 ± 0.36 ms (n = 13)
EPSC-IEI distribution τ_1_	20.4 ± 2.4 ms (n = 15)	27.1 ± 2.2 ms (n = 13)
EPSC-IEI distribution τ_2_	180.7 ± 24.3 ms (n = 15)	148.7 ± 17.2 ms (n = 13)
EPSC-IEI distribution A_1_	68.2% ± 1.44% (n = 15)	63.7% ± 3.0% (n = 13)
EPSC-IEI distribution A_2_	31.8% ± 1.44% (n = 15)	36.3% ± 3.0% (n = 13)
Phase angle EPSCs versus LFP, theta range	NA	321° ± 12° (n = 13)
Action potentials per burst	NA	3.3 ± 0.9 (n = 3)
Phase angle action potentials versus LFP, theta range	NA	284° ± 21° (n = 3)
Phase angle action potentials versus LFP, gamma range	NA	340° ± 21° (n = 3)

n indicates the number of experiments. ND, not determined (limited number of traces); NA, not applicable; IEI, interevent interval. See also Figure S2.

a The supratheta range mean frequency of EPSCs reflects the occurrence of EPSC bursts.

b Parameters that are statistically different between the two recording conditions (p < 0.05).

### Synaptic Excitation of GCs In Vivo

**Figure 3:**
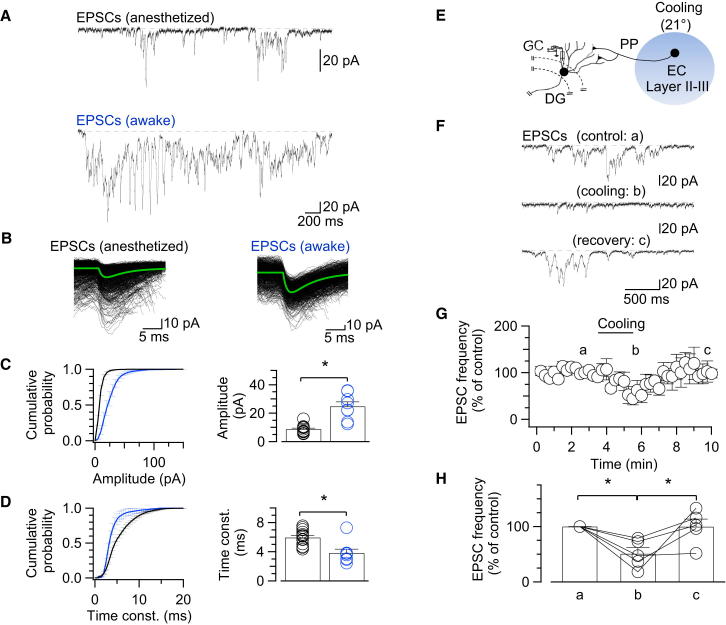
Hippocampal GCs In Vivo Are Exposed to Barrages of Fast EPSCs Originating in the Entorhinal Cortex (A) Representative trains of EPSCs recorded from dentate gyrus GCs in anesthetized (top) and awake (bottom) rats at –70 mV. (B) EPSCs detected by a deconvolution-based method, aligned and superimposed according to the detection point. Data from anesthetized (left) and awake (right) rats are shown. Black traces indicate individual EPSCs, green trace represents the average EPSC (985 and 844 superimposed traces, respectively). (C) Cumulative probability distribution (left) and summary bar graph (right) of EPSC peak amplitude. ^∗^p < 0.05. (D) Cumulative probability distribution (left) and summary bar graph (right) of EPSC decay time constant. ^∗^p < 0.05. Color code in (C) and (D): black, anesthetized (15 cells); blue, awake animals (13 cells). (E) Schematic illustration of focal thermal inactivation of the entorhinal cortex. EC, entorhinal cortex; PP, perforant path. (F) Representative recording of EPSCs at –70 mV holding potential in a GC, before (“control”), during (“cooling”), and after (“recovery”) cooling of the ipsilateral EC. (G) Plot of EPSC frequency against time during cooling. Average data from five cells are shown. Labels (a), (b), and (c) indicate time points of traces shown in (F). (H) Summary of EPSC frequency before, during, and after cooling, normalized to control values. ^∗^p < 0.05. Bar graphs represent mean ± SEM, circles indicate data from individual cells. Data from the same cell are connected by lines. Data in (E)–(H) were obtained from anesthetized rats (five cells). See also Figure S3.

A key prediction of the excitation model of theta-gamma oscillations (Figure 1B) is that GCs should receive phasic excitatory synaptic input. We therefore examined EPSCs under voltage-clamp conditions at a holding potential of –70 mV, close to the reversal potential of GABA_A_R-mediated IPSCs (Figures 3A–3D; Table 1). EPSC detection (Pernía-Andrade et al., 2012) followed by kinetic analysis revealed that GCs in vivo in both anesthetized and awake rats were exposed to a high-frequency excitatory phasic input (Figures 3A and 3B). On average, the peak amplitude of individual EPSCs was 8.8 ± 0.7 pA in anesthetized rats and 21.3 ± 2.4 pA in awake rats (15 and 13 cells, respectively; p < 0.0001; Figure 3C). Furthermore, the EPSC mean decay time constant was 5.95 ± 0.26 ms in anesthetized rats and 3.84 ± 0.36 ms in awake rats (p < 0.01; Figure 3D). Finally, analysis of EPSC timing revealed that interevent intervals (IEIs) were distributed according to two exponential components, with time constants of τ_1_ = 20.4 ± 2.4 ms and τ_2_ = 180.7 ± 24.3 ms in anesthetized rats and τ_1_ = 27.1 ± 2.2 ms and τ_2_ = 148.7 ± 17.2 ms in awake rats (Figure S2). Thus, EPSCs were not randomly generated but were clustered in bursts. Charge recovery analysis revealed that fast EPSCs accounted for 83% ± 3% of the total activity at –70 mV (Experimental Procedures). In conclusion, GCs received a massive excitatory input, which was to a large extent caused by trains of fast EPSCs.

To determine the source of EPSCs in GCs, we attempted to suppress the presynaptic neurons by focal thermoinactivation using a micro-Peltier element (Figure 3E). Focal thermoinactivation of the ipsilateral entorhinal cortex significantly and reversibly reduced the frequency of EPSCs to 51% ± 11% of control value (five cells in anesthetized rats; p < 0.05; Figures 3F–3H), without significant changes in EPSC amplitude or kinetics (3%–8% change; p > 0.1). Thus, a major component of EPSC activity in GCs appeared to originate in the ipsilateral entorhinal cortex (Bragin et al., 1995, Chrobak and Buzsáki, 1998).

To determine the identity of the types of receptors involved in the activity, we further attempted to block the synaptic events by a selective antagonist via local perfusion (Figure S3A). Local application of 10 μM CNQX in the dentate gyrus reduced synaptic activity to 29.7% ± 19.2% of control value (four cells in anesthetized rats; p < 0.05; Figure S3B–S3D). Thus, a major fraction of synaptic activity at –70 mV was mediated by AMPA-type glutamate receptors. Taken together, the results suggest that GCs in vivo were exposed to barrages of fast AMPAR-mediated EPSCs, which were primarily relayed from the entorhinal cortex.

### Theta-Coherent EPSCs in GCs In Vivo

**Figure 4:**
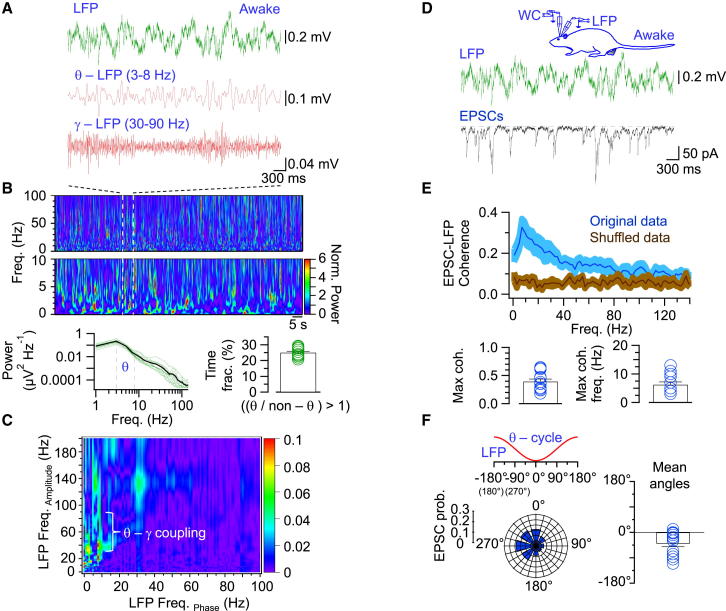
Theta-Coherent Synaptic Excitation in Hippocampal GCs in Awake Rats In Vivo (A) Recording of LFP in an awake rat. Top: minimally filtered trace (150 Hz low-pass; green); middle: same trace filtered in the theta range (3–8 Hz; red); bottom: same trace filtered in the gamma range (30–90 Hz; red). (B) Top: frequency-time representation of normalized power of the unfiltered LFP recording (same experiment shown in A) on a compressed timescale. Data from a representative experiment (top: 0–100 Hz frequency range; middle: 0–10 Hz frequency range; dashed lines indicate the time interval corresponding to the traces in A) are shown. Normalized power is color coded (calibration bar on the right). Notice the presence of multiple theta and gamma power peaks along 2 min of continuously recorded LFP activity. Bottom left: average power spectrum (green, single experiments; black, average; vertical dashed lines indicate the theta band). Bottom right: fraction of experimental time in which the ratio of theta to nontheta power is >1. Note that theta rhythm is a predominant form or activity under our experimental conditions. (C) Average cross-frequency coherence analysis of LFP activity. Note the existence of coupling between LFP amplitude envelope at 30–90 Hz and LFP phase at 3–10 Hz. Coupling strength is color coded (calibration bar on the right). (D) Simultaneous recording of EPSCs and LFP in awake rats (same experiment as in A). EPSCs were recorded in the WC voltage-clamp configuration at –70 mV, close to the GABA_A_R reversal potential. Green, LFP recording; black, WC recording. (E) Top: average coherence between EPSCs and the LFP. To evaluate the statistical significance of coherence, we compared the original data (blue) to shuffled data (brown). Shaded areas indicate SEM. Control data were significantly different from shuffled data (p < 0.05; Kruskal-Wallis test). Bottom: summary bar graph of maximum coherence and corresponding frequency. (F) Phase relationship between EPSCs and LFP during a theta cycle. Left: polar plot illustrates distribution of onset points of EPSCs detected by deconvolution. As the LFP recording electrode was located in the molecular layer of the dentate gyrus, the trough of the theta cycle was taken as a reference point for phase measurement (0°). Distribution of angular deviations differed significantly from a uniformity (p < 0.005; Rayleigh test; see Experimental Procedures). Data from a representative experiment are shown. Right: angular lag of EPSCs to LFP trough. All experiments were performed in awake rats (13 simultaneous LFP-WC recordings; LFP analysis in A–C, combined LFP-WC analysis of D–F). In summary graphs, bars indicate mean ± SEM, circles represent data from individual experiments. See also Figures S4 and S5.

Another prediction of the excitation model of theta-gamma oscillations (Figure 1B) is that EPSCs should be coherent with the LFP. To test this prediction, we made simultaneous recordings of EPSCs and the LFP from the dentate gyrus in awake rats (Figure 4; Table 1). We first examined the basic properties of the LFP in the dentate gyrus. Analysis of the power spectrum revealed that the LFP contained both theta and gamma components (Figures 4A and 4B). In awake rats, theta activity was a highly abundant form of network activity; the ratio of theta to nontheta power exceeded one in 25.1% ± 0.8% of the experimental time (13 experiments; Figure 4B). Furthermore, cross-frequency coherence analysis demonstrated that gamma and theta oscillations were nested (Figure 4C), as reported previously (Bragin et al., 1995, their Figure 1). Finally, LFP power in the theta range was reduced by thermoinactivation of the ipsilateral entorhinal cortex (Figure S5), consistent with the results of previous lesion experiments (Bragin et al., 1995).

We then examined the relations between EPSC and LFP signals (Figures 4D–4F). Analysis of the coherence between the two signals revealed a high level of coherence in the theta frequency range but a low coherence in the gamma frequency band (Figure 4E). The main peak had a mean coherence of 0.40 ± 0.04, corresponding to a frequency of 6.2 ± 1.0 Hz (13 cells in awake rats; Figure 4D). Control analysis with shuffled data showed that the coherence was significant (p < 0.05; Figure 4E). Furthermore, phase analysis demonstrated that EPSCs were significantly phase locked to theta cycles of the LFP (p < 0.005). The angular lag for the theta activity was –39° ± 12° (321°; 13 cells), implying that EPSCs coincided with the descending phases and the troughs of the theta oscillations (Figure 4F). Consistent with these results, EPSC power spectra showed a peak at theta frequency, with a maximum at 4.3 ± 0.3 Hz in anesthetized rats and 6.1 ± 0.4 Hz in awake rats (15 and 13 cells, respectively; Figures S6A and S6B; see Klausberger et al., 2003). Furthermore, autocorrelation analysis of EPSC traces revealed regular peaks at a mean period of 204.6 ± 27.8 ms in anesthetized and 179.1 ± 18.8 ms in awake rats (Figures S6C and S6D). Taken together, these results indicate that EPSCs represent a global synaptic input signal, which is spatially coherent over the dentate gyrus and mainly operates at theta frequency. While the high coherence in the theta frequency range is consistent with the excitation model, the lower coherence in the gamma frequency range seems inconsistent with this model.

### Gamma-Coherent IPSCs in GCs In Vivo

**Figure 5:**
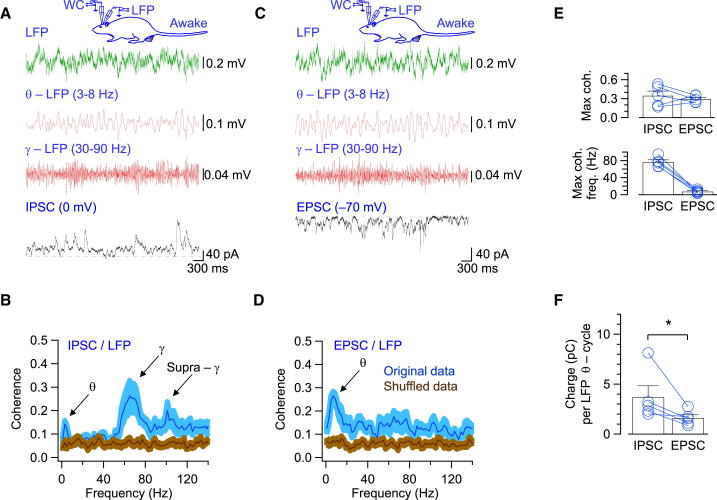
Gamma-Coherent Synaptic Inhibition in Hippocampal GCs In Vivo (A) Simultaneous recording of IPSCs and LFP. IPSCs were recorded in the WC voltage-clamp configuration at 0 mV, close to the reversal potential of AMPAR-mediated currents. Green, LFP recording; red, same LFP trace filtered in the theta (second trace from top) and gamma (third trace from top) frequency range; black, WC recording. (B) Average coherence between IPSCs and LFP. Note the presence of a peak in the gamma frequency range and two additional peaks in theta and supragamma frequency range (arrows). (C) Simultaneous recording of EPSCs and LFP. EPSCs were recorded in the WC voltage-clamp configuration at –70 mV, close to the GABA_A_R reversal potential. Color code is the same as in (A). (D) Average coherence between EPSCs and LFP. Note the presence of a peak at theta frequency (arrow). To evaluate the statistical significance of coherence in (B) and (D), we compared the original data (blue) to shuffled data (brown). Shaded areas indicate SEM. Control data were significantly different from shuffled data (p < 0.05). Data in (A) and (C) were recorded from the same individual cell; data in (B) and (D) were obtained from the same population of neurons (five cells with at least one cycle of sequential EPSC-IPSC-EPSC measurements). (E) Maximal coherence (top) and corresponding frequency (bottom) for EPSCs and IPSCs. (F) Total charge of EPSCs and IPSCs per theta cycle. Note that the ratio of inhibitory to excitatory charge was approximately constant, indicating that excitation and inhibition were balanced. Bars indicate mean ± SEM, circles represent data from individual experiments. Data points from the same experiment are connected by lines. EPSC data shown in (D) and (E) were also included in Figure 4E. All experiments were performed in awake rats (five cells). See also Figures S4 and S6.

If EPSCs are strongly theta coherent but only weakly gamma coherent, what are the synaptic mechanisms underlying gamma oscillations in the dentate gyrus (Bragin et al., 1995)? To address this question, we recorded IPSCs in GCs and examined the coherence with the LFP in awake rats (Figure 5). IPSCs were isolated under voltage-clamp conditions at a holding potential of 0 mV, close to the reversal potential of AMPAR-mediated currents. Surprisingly, the frequency dependence of coherence of IPSCs was markedly different from that of EPSCs. Analysis of coherence between the IPSC signal and the LFP indicated a highly significant peak in the gamma frequency range (five cells in awake rats; p < 0.05). The main peak had a mean coherence of 0.35 ± 0.07, corresponding to a frequency of 76.2 ± 5.2 Hz. Additional coherence peaks of lower amplitude were present in both the theta (coherence 0.14 ± 0.03, frequency 3.4 ± 0.4 Hz) and the supragamma frequency ranges (coherence 0.26 ± 0.05, frequency 101 ± 8 Hz; Figure 5B). Thus, IPSC signals were coherent to the LFP primarily in the gamma frequency band. To compare the coherence of IPSCs and EPSCs with the LFP in the same cells, we recorded EPSCs under conditions in which membrane potentials were alternated between 0 mV and –70 mV (Figures 5C and 5D). For EPSCs, the coherence showed a peak in the theta frequency range, demonstrating that gamma-coherent IPSCs and theta-coherent EPSCs can be recorded in the same cell (Figure 5E). Moreover, cross-frequency coherence analysis revealed that theta-gamma components of IPSCs and EPSCs were differentially coupled to the LFP theta phase (Figure S4).

To further address whether IPSCs and EPSCs were correlated in amplitude, we determined the total charge per theta cycle (∼200 ms; Figure 5F). Although both excitatory and inhibitory synaptic charges (as obtained by integration of EPSCs and IPSCs) showed substantial variability among individual cells, their ratio was approximately constant (2.3 ± 0.3), indicating that excitation and inhibition were well balanced. In conclusion, theta-gamma oscillations in the dentate gyrus are mediated by a combination of theta-coherent excitation and gamma-coherent inhibition. The balance of excitation and inhibition may explain the tight association of theta and gamma rhythm in vivo (Bragin et al., 1995). Thus, our results suggest a revised model of theta-gamma oscillations in the dentate gyrus (Figure 1C), which differs critically from the previous models (Figures 1A and 1B).

### Theta-Gamma-Modulated Synaptic Currents Set Action Potential Timing

**Figure 6:**
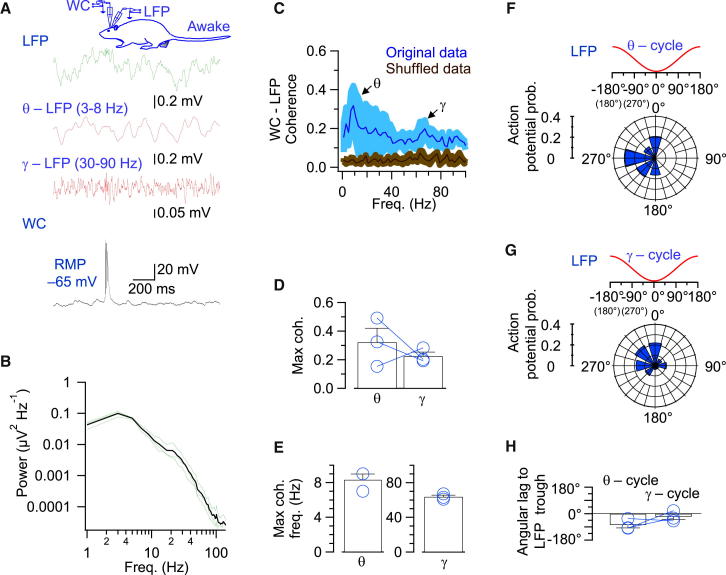
Action Potentials in GCs Are Phase Locked to the Local LFP in Both Theta and Gamma Frequency Bands (A) Simultaneous recording of LFP and WC membrane potential in awake condition. Top: minimally filtered trace (150 Hz low-pass; green); middle (red): same trace filtered in the theta (second trace from top) and gamma range (third trace from top); bottom: WC membrane potential (black). (B) Power spectrum of the LFP in the three experiments in which GCs generated action potentials in awake condition (see Figure 2E). Green curves, single experiments; Black curve, average. (C) Coherence between LFP and WC membrane potential recording (including action potentials). Blue, original data; brown, shuffled data. Shaded areas indicate SEM. Control data were significantly different from shuffled data (p < 0.05). Note the presence of coherence peaks in both theta and gamma frequency band. (D) Summary bar graph of maximum coherence. (E) Summary bar graph of corresponding frequency in theta (left) and gamma (right) frequency range. (F and G) Phase relationship between action potential/burst onset and theta cycle (F) or gamma cycle (G) in one of the three firing GCs in awake condition. (H) Angular lag between action potential/burst onset and LFP trough in awake rats for theta and gamma frequency oscillations. Note that GCs fired in the descending phases of theta and gamma cycles, respectively. See also Figures S4 and S7.

What is the function of a coherent theta-gamma-modulated synaptic signal in the dentate gyrus network? One possibility is that synaptic currents provide a reference signal for temporal encoding, in which the exact time interval between action potentials and synaptic currents encodes information (Buzsáki and Draguhn, 2004). Temporal coding may be highly important in the dentate gyrus, where action potential frequency is very low (Figure 2) and therefore rate codes cannot be used. To test this idea, we recorded action potential activity in GCs under current-clamp conditions in awake rats (Figure 6; Table 1). In the subpopulation of firing GCs, analysis of coherence between membrane potential (including action potentials) and LFP revealed significant peaks at both theta and gamma frequencies (coherence 0.32 ± 0.10, frequency 8.3 ± 0.7 Hz, and coherence 0.23 ± 0.03, frequency 63.7 ± 1.8 Hz respectively; Figures 6C–6E). Furthermore, action potentials were significantly phase locked to both theta and gamma cycles of the LFP (p < 0.002 and p < 0.05, respectively), with action potentials frequently occurring in the descending theta-gamma phases (Figures 6F–6H). Reverse analysis by action potential-triggered LFP averaging corroborated these conclusions (Figure S7). These results are consistent with the idea that theta-gamma-modulated synaptic currents provide a reference signal for temporal encoding of information in the dentate gyrus.

## Discussion

The present results solve a paradox in our current understanding of the mechanisms of theta-gamma oscillations in the dentate gyrus: that gamma activity appears to primarily rely on inhibition (Bartos et al., 2007) but is reduced by entorhinal lesions that will mainly compromise excitation (Bragin et al., 1995). We show that EPSCs in GCs are coherent with the LFP in the theta frequency range but to a much smaller extent in the gamma frequency range. Conversely, IPSCs are more coherent in the gamma than in the theta frequency band. Thus, two spectrally and mechanistically distinct rhythmic signals coexist in the dentate gyrus, with theta activity mainly relayed from the entorhinal cortex via excitation and gamma activity generated by local inhibition (Figure 1C).

### Mechanisms of Theta Generation in the Dentate Gyrus

The classical model of generation of theta oscillation assumes that cholinergic input from the medial septum/diagonal band plays a critical role in theta generation (“atropine-sensitive theta”; Stewart and Fox, 1990). Additionally, disinhibition via local interneurons may contribute to the theta rhythm (Freund and Antal, 1988). Finally, intrinsic oscillatory mechanisms may be involved (Goutagny et al., 2009). Our results demonstrate that GCs in vivo are exposed to massive functional glutamatergic input from the entorhinal cortex. EPSCs are theta coherent with the LFP, suggesting that they provide a major contribution to the rhythm. Direct cholinergic input on GCs plays only a minor role, since a main portion of excitatory activity is blocked by CNQX (Figure S3). Furthermore, disinhibition may not convey a major component of theta, since IPSCs are only weakly theta coherent (Figure 5). In contrast, our results suggest that a major theta component is relayed from the entorhinal cortex (Figure 1C).

### Mechanisms of Gamma Rhythm

Several lines of evidence suggest that GABAergic interneurons, especially fast-spiking, parvalbumin-expressing subtypes, play a key role in the generation of gamma oscillations in various regions of the brain (Bartos et al., 2007, Buzsáki and Wang, 2012, Varga et al., 2012). In the dentate gyrus, however, both the power and frequency of gamma oscillations are reduced by chronic lesions of the entorhinal cortex (Bragin et al., 1995). Our results show that EPSCs, although they have high-frequency components, are only weakly gamma coherent with the LFP. Thus, a scenario in which the gamma rhythm is relayed from the entorhinal cortex to the dentate gyrus in a 1:1 manner seems unlikely. In contrast, IPSCs show a high degree of gamma coherence. Thus, whereas the theta rhythm is mainly relayed from the entorhinal cortex via excitation, the gamma rhythm is primarily generated by inhibition, most likely locally by GABAergic interneurons (Bartos et al., 2007, Buzsáki and Wang, 2012; Figure 1C). Although previous studies showed that perisomatic inhibition markedly contributes to gamma oscillations in vitro (Mann et al., 2005), our results provide a direct demonstration that this is also the case in vivo in awake animals. For both theta and gamma activity, cholinergic or GABAergic inputs from the septum may exert an indirect modulatory role via innervation of entorhinal cortex pyramidal neurons or dentate gyrus GABAergic interneurons.

### Spatially Separated Synaptic Mechanisms Control Spike Timing in GCs

Our results reveal a division of labor between excitatory and inhibitory synapses in the generation of nested theta-gamma oscillations: EPSCs are mainly theta coherent, whereas IPSCs are gamma coherent. Furthermore, our findings demonstrate that action potentials in GCs are phase locked to nested theta-gamma LFP oscillations. These data suggest that the compound EPSC-IPSC signal may work as a highly efficient reference signal for temporal encoding in dentate gyrus GCs. How is precise spike timing achieved under these conditions? Excitatory and inhibitory synapses are differentially distributed along the somatodendritic axis of GCs. While excitatory input from the perforant path is directed to the inner and outer molecular layer, a major portion of inhibitory synapses is located perisomatically (Freund and Buzsáki, 1996). Thus, excitatory and inhibitory synaptic events will be differentially affected by cable filtering (Schmidt-Hieber et al., 2007, Krueppel et al., 2011). Cable modeling of dentate gyrus GCs indicated that the dendrosomatic transfer impedance is highly frequency and location dependent (Carnevale et al., 1997, Schmidt-Hieber et al., 2007, Krueppel et al., 2011). Thus, proximal inputs can provide signals in the gamma frequency range, whereas distal inputs may provide signals with slower frequency characteristics (e.g., theta). A mechanism for theta-gamma oscillations based on spatially separated synaptic inputs may be particularly useful in dentate gyrus GCs, in which intrinsic mechanisms of rhythmic membrane potential oscillations appear to be absent (Krueppel et al., 2011).

### Implications for Information Processing in the Dentate Gyrus

Previous work suggested two different coding schemes in the brain: rate coding and temporal coding. If action potential frequency in dentate gyrus GCs is low in several conditions, as our findings suggest (Figure 2), rate coding schemes will be very inefficient. In contrast, temporal coding schemes may be more effective. Our results show that the onset of action potentials in GCs is phase locked to the descending phase of the theta and gamma phase in the LFP (Figure 6). This suggests that action potentials are generated at temporally precise time points in the theta-gamma cycle, defined by the temporally modulated pattern of synaptic currents. Thus, our results are consistent with the idea that dentate gyrus GCs use a temporal coding scheme in both theta and gamma frequency bands.

Two major network functions have been attributed to the dentate gyrus: pattern separation (Leutgeb et al., 2007) and grid-to-place code conversion (de Almeida et al., 2009b). Theta-gamma-modulated synaptic currents will support these functions in multiple ways. Models of dentate gyrus networks suggest that rhythmic oscillations, particularly gamma oscillations, may be involved in the selection of cells that receive the highest excitation level by a “winner takes all” mechanism (de Almeida et al., 2009a, de Almeida et al., 2009b). This mechanism would be expected to amplify subtle differences between input patterns, which would generate, for example, pattern separation. Furthermore, this mechanism would amplify small differences in peaks of grid cell firing, resulting in a conversion from grid-to-place codes. Thus, the oscillatory structure of EPSCs and IPSCs may represent a framework for both pattern separation and grid-to-place code conversion in the dentate gyrus.

### Functional Consequences for GC Output

The firing of hippocampal GCs in vivo previously was controversial. Early studies indicated high-frequency activity of GCs in the center of place fields (Jung and McNaughton, 1993, Skaggs et al., 1996, Leutgeb et al., 2007) and during working memory tasks (Wiebe and Stäubli, 1999). In contrast, more recent work indicated that GCs in vivo are largely silent (Alme et al., 2010). Our results demonstrate that morphologically identified GCs in awake rats fire at low frequency. However, when GCs generate spikes, they preferentially fire in bursts. Both the negative resting potential and the coexistence of firing and silent GCs are consistent with the idea that bursting does not represent an artifact of WC recording or a pathophysiological event. Thus, mature GCs in awake animals may primarily use a sparse burst coding mechanism for representation of information (reviewed by Lisman, 1997). Low-frequency bursting activity has major implications for GC output via the mossy fiber system. In combination, the low frequency of spiking and the high proportion of bursts will maximize facilitation at hippocampal mossy fiber synapses, the sole output synapses from dentate gyrus GCs (Salin et al., 1996, Toth et al., 2000, Henze et al., 2002).

Together with previous results, our findings suggest that two highly nonlinear steps in series govern signal flow from the dentate gyrus to the CA3 region. In the first step, pattern separation promoted by gamma oscillations (de Almeida et al., 2009a, de Almeida et al., 2009b) extracts the differences between input patterns. In the second step, burst amplification of mossy fiber transmission generates a highly efficient output onto CA3 pyramidal neurons. This enchainment of two highly nonlinear processes ensures that novel information is selectively relayed to the CA3 region, where it can be used to initiate the efficient storage in CA3–CA3 pyramidal neuron synapses via heterosynaptic potentiation (Kobayashi and Poo, 2004, Bischofberger et al., 2006).

## Experimental Procedures

### Preparation

Patch-clamp recordings were made from morphologically identified mature dentate gyrus GCs of the dorsal hippocampus in vivo, using 28- ± 1-day-old Wistar rats of either sex. Experiments followed previous protocols (Margrie et al., 2002, Lee et al., 2006, Lee et al., 2009), although extensive modification was necessary to account for the deep location of hippocampal GCs. All experiments were carried out in strict accordance with national and European guidelines for animal experimentation. Protocols were approved by the Bundesministerium für Wissenschaft und Forschung of Austria (BMWF-66.018/0008-II/3b/2010). Animals were maintained under light (7 a.m.–7 p.m.) and dark (7 p.m.–7 a.m.) cycle conditions, and experiments were performed from 3 p.m. to 10 p.m. Animals of a litter were separated at postnatal day 21, after which they were kept under single animal per cage conditions until the day of the experiment.

Animals were anesthetized by intraperitoneal (i.p.) injection of 0.3 mg/kg medetomidine (Pfizer), 8 mg/kg midazolam (Roche), and 0.01 mg/kg fentanyl (Janssen-Cilag; Lee et al., 2006) in the experiments on anesthetized rats or by 80 mg/kg ketamine (Intervet) and 8 mg/kg xylazine (Graeub) for experiments on awake animals (all doses per kg body weight). For craniotomy, rats were mounted in a stereotaxic frame (David Kopf Instruments), in which the head of the animal was fixed with a pair of ear bars and a perpendicular tooth bar. Measurements were obtained from the dorsal hippocampus, a region specifically involved in spatial coding and memory. Stereotaxic coordinates (anterioposterior [AP] measured from bregma; lateral [L] specified from midline; dorsoventral [DV] from surface of the brain) were set according to Paxinos and Watson (1998), after appropriate scaling from adult to postnatal day 28 skull and brain geometry. One or two craniotomies were made to target the dorsal hippocampus (AP ∼3.5 mm, L ∼2.5 mm) of the right or left hemisphere for WC and LFP recordings and the ipsilateral entorhinal cortex (AP ∼8.9 mm, L ∼3.7 mm) for insertion of a micro-Peltier element. In addition, up to five fixation holes (∼1 mm diameter) were drilled into the skull (two contralateral, two occipital, and one frontal). Within the craniotomy windows, the dura mater was cut and removed using iridectomy scissors and Dumont 5 forceps (FST). Craniotomy windows were repeatedly superfused with physiological saline solution (135 mM NaCl, 5.4 mM KCl, 1.8 mM CaCl_2_, 1 mM MgCl_2_, and 5 mM HEPES [pH = 7.2]). A custom-made fixation ring (GFK fiberglass, R&G Faserverbundwerkstoffe) was attached to the skull via microscrews inserted into the fixation holes and additionally fixed on the skull using dental cement (Paladur; Heraeus). Ear and tooth bars were removed after the dental cement was fully cured. Thus, the rat was stably head fixed via the fixation ring.

For recordings from anesthetized rats, animals were left in the stereotaxic frame and the medetomidine + midazolam + fentanyl anesthesia was continued by additional injections of 25% of the initial dose at ∼50 min intervals. Cardiovascular and respiratory functions were continuously monitored by measuring heart rate and arterial O_2_ saturation using a PulseSense monitoring system (PulseSense Vet, medair). O_2_ gas was applied continuously via the ventilation mask. Typically, heart rate was 250–300 beats min^−1^ and arterial O_2_ saturation was >98%. Body temperature was continuously monitored by a rectal thermometer and maintained at 37°C ± 0.5°C by placing the animal on a heating pad.

For experiments on head-fixed, fully awake rats, animals were remounted in a second frame above a spherical treadmill (air-supported polystyrol ball with 300 mm diameter; Jetball, PhenoSys; see Dombeck et al., 2007). In this system, animals were able to groom, rest, or run, with maximal linear velocities of 40 cm s^−1^. Rats were allowed to recover from anesthesia and adapt to the recording device for at least 3 hr. The insertion of the recording electrodes was performed under a light and brief inhalation anesthesia, applying 0.2%–0.4% isoflurane (Forane; Abbott) via a ventilation mask for <5 min. Anesthesia was terminated immediately after the WC configuration was established, and data acquisition was started ∼10 min later. Analgesia was ensured by i.p. application of 50 mg/kg metamizole (Sanofi-Aventis; in strict accordance with animal regulations). In awake animals, all sensors were removed to minimize stress. Vigilance of animals was judged by high muscle tone, movement of whiskers, tail, and limbs, the presence of postural reactions, and locomotor patterns. Animals were able to move on the spherical treadmill freely but characteristically showed a low level of motor activity under our conditions, with long periods of immobility/lingering and short periods of movement, as expected during exploration of a relatively new environment (Whishaw and Kolb, 2005). The total recording time was 5–30 min (including periods of both immobility and moderate motor activity). Robust theta and gamma activity was recorded in the LFP under these behavioral conditions. However, our theta peak frequency corresponded to the lower part of the previously defined theta frequency range, presumably due to the inclusion of both immobility and moderate motor activity periods in our analysis (Bland, 1986, Buzsáki, 2002).

### Patch-Clamp Recording from GCs In Vivo

Pipettes for both WC and LFP recording were fabricated with a Brown-Flaming micropipette puller (either P-97 or P-1000; Sutter Instrument), using 1 mm outer diameter and 0.5 mm inner diameter borosilicate glass capillaries (Hilgenberg). Pipettes used for patch-clamp recording had tip resistances of 4–7 MΩ. For current-clamp experiments, pipette solution contained 134 mM K-gluconate, 2 mM KCl, 10 mM EGTA, 2 mM MgCl_2_, 2 mM Na_2_ATP, 10 mM HEPES, and 3 mg ml^−1^ biocytin (pH adjusted to 7.28 with KOH). For voltage-clamp experiments with EPSCs, a pipette solution containing 134 mM K-methanesulfonate, 2 mM KCl, 10 mM EGTA, 2 mM MgCl_2_, 2 mM Na_2_ATP, 10 mM HEPES, 3 mg ml^−1^ biocytin, and 5 mM QX-314 was used. Finally, for voltage-clamp experiments in which both EPSCs and IPSCs were measured, pipette solution contained 120 mM Cs-methanesulfonate, 20 mM KCl, 10 mM EGTA, 2 mM MgCl_2_, 2 mM Na_2_ATP, 10 mM HEPES, 3 mg ml^−1^ biocytin, and 5 mM QX-314; in these experiments, tip filling was made with K-methanesulfonate-containing solution. Osmolarity of internal solutions was set to 310 ± 5 mOsm by addition of sucrose as required.

Patch pipettes were gently advanced in the vertical (DV) direction, targeting the dentate gyrus GC layer (AP –3.5 to –5.0 mm, L 2.5 to 3.0 mm, and DV –2.9 to –3.2 mm; Paxinos and Watson, 1998). Positive pressure (500–900 mbar) was applied to the pipette interior while crossing the neocortex and corpus callosum, until ∼200 μm above the target zone. Subsequently, the pressure was gradually reduced to ∼20 mbar. Finally, blind WC recordings were obtained, based on changes in current amplitudes in response to a 10 mV test pulse (Castañeda-Castellanos et al., 2006, Margrie et al., 2002, Lee et al., 2006, Lee et al., 2009). Only cells with initial seal resistance >3 GΩ were included in this study. The integrity of the seal was verified by formation of an outside-out patch during withdrawal of the pipette after completion of the experiment. In current-clamp experiments, voltage measurements were made without holding current injection. In voltage-clamp recordings, the holding potential was set either to –70 mV for EPSC recording or to 0 mV for IPSC recording. As recordings were started ∼10 min after the whole-cell configuration was obtained, sufficient time for clearance of K^+^ or Cs^+^ that might have accumulated during the patch-clamp procedure was ensured.

Pipettes used for LFP recording had tip resistances of 1–3 MΩ. Pipettes were filled with physiological saline solution containing 3 mg ml^−1^ biocytin. Pipettes were gently inserted, with a 20° oblique angle, in the AP direction, targeting the molecular layer of the dorsal hippocampus (AP –5.6 mm, L 3.4 mm, DV –3.4 mm). Positive pressure (100–200 mbar) was applied to avoid pipette plugging. A common reference electrode (Ag/AgCl) was placed on the skull close to the craniotomy windows. Both the WC recorded neuron and the LFP electrode location were visualized by post hoc biocytin labeling, using 3,3′-diaminobenzidine (DAB) as chromogen. To minimize spurious labeling, we immediately terminated suboptimal WC recordings by pipette removal and only a single LFP recording pipette was inserted per animal. The average distance between WC and LFP pipette tips was 1.26 ± 0.10 mm (five anesthetized and eight awake rats).

For focal thermoinactivation experiments (Figure 3E; Figure S5), a micro-Peltier element was used. The device was inserted into the ipsilateral entorhinal cortex in the parasagittal plane, at a 10° oblique angle to the transverse plane; the tip was placed at 8.6–9.2 mm AP, 3.4–4.0 mm L, and 1.8–2 mm DV. Tip location was verified by post hoc histology in all cases. The device was assembled from a Peltier element (ETH-127-10-13-S-RS; Global Component Sourcing) connected to a DC power supply (1–25 W). The cold side of the Peltier element was connected to a customized copper clamp (length ∼2.5 cm), which held a silver wire (length ∼3 cm; cross-sectional area 0.79 μm^2^). To avoid electrical contact with brain tissue, we covered the silver wire with nail polish. The warm side of the Peltier element was connected to a water-cooling system (Basic LC Plus PC water cooling set 800654 with adaptor Graph-O-Matic v. 3.0; Innovatek). Control measurements with microthermistors (diameter 0.4 mm) revealed that the cooling effect was local, with ∼10°C temperature drop in the entorhinal cortex but <1°C in the hippocampus. Cooling is expected to reduce both action potential initiation and transmitter release but would not be expected to completely suppress it, consistent with our experimental observations (Figures 3F–3H). In contrast to the marked effects on EPSC frequency, thermoinactivation led to only minimal changes in holding current (<10 pA) or input resistance of GCs.

For application of synaptic blockers, a double barrel microinjection system was used (Figure S3A). The barrels (fabricated from 0.4 mm outer diameter injection needles) were attached in parallel to the recording pipette. Barrel outlets were separated from the tip of the pipette by <1 mm, and the oblique side was directed toward the recording pipette to ensure application of drugs to the recorded cell. The barrels were connected to two independent perfusion pumps (Aladdin-1000, WPI) and perfused at a total rate of 8 μl min^−1^. 6-cyano-7-nitroquinoxaline-2,3-dione (CNQX) was from Biotrend; other chemicals were from Sigma-Aldrich or Merck.

### Biocytin Labeling

For analysis of neuron morphology after recording (Figure 2A), brains were fixed >24 hr in 2.5% paraformaldehyde, 1.25% glutaraldehyde, and 15% saturated picric acid in 100 mM phosphate buffer (PB; pH 7.35). The hemisphere containing the recorded cell was cut into 200-μm-thick parasagittal slices. After fixation, slices were washed, incubated in 2% hydrogen peroxide, and shock frozen in liquid nitrogen. Subsequently, the tissue was treated with PB containing 1% avidin-biotinylated horseradish peroxidase complex (ABC; Vector Laboratories) overnight at 4°C. Excess ABC was removed by several rinses with PB, before development with 0.05% 3,3′-diaminobenzidine tetrahydrochloride and 0.01% hydrogen peroxide. Subsequently, slices were rinsed in PB several times and embedded in Mowiol (Roth). All GCs reported in this paper were rigorously identified as mature GCs, based on the location of the soma in the GC layer, the complex dendritic arbor, the presence of dendritic spines in high density, and the labeling of mossy fiber axons and boutons (Lübke et al., 1998, Schmidt-Hieber et al., 2004).

### Data Acquisition and Analysis

In total, recordings were obtained from 46 rigorously identified GCs in vivo. Synaptic potentials, currents, and LFPs were recorded using an EPC10 double patch-clamp amplifier (HEKA). Signals were low-pass filtered at 10 kHz (Bessel) and sampled at 20 kHz using Patchmaster software. The access resistance was 43.3 ± 1.2 MΩ (range: 25.0–57.5 MΩ; 46 cells).

Baseline values in both current- and voltage-clamp recordings were determined in 100 ms temporal windows in which the variance was low (within tenth percentile of the entire variance distribution). To measure the apparent membrane time constant (τ_m_), hyperpolarizing voltage changes during –50 pA current pulses were fit with a biexponential function; τ_m_ was approximated from the slow component of the fit. To measure the input resistance, we plotted membrane potential at the end of a 1 s pulse against injected current and fitted by linear regression. To obtain frequency-current curves, we computed the average instantaneous action potential frequency from responses to 1 s depolarizing current pulses.

EPSCs were detected by a deconvolution-based algorithm (Pernía-Andrade et al., 2012). This procedure is particularly suitable for analysis of synaptic events in vivo, because of its high temporal resolution. Briefly, experimental traces were converted into a series of delta-like functions, the local maxima of which were used for event detection and alignment. Temporal resolution was set to 1 ms (1 kHz). The amplitude criterion for detection was set to 4.3 × SD of baseline noise, corresponding to a false positive rate of 0.17 points per second (Pernía-Andrade et al., 2012). After detection, kinetics and temporal structure of events were analyzed using scripts written in Igor Pro (version 6.22A; Wavemetrics). Charge recovery analysis was performed by calculating the ratio of the sum of integrals under all the detected synaptic events divided by the integral under the total trace.

For analysis and display, synaptic signals were additionally filtered using a digital 1 kHz low-pass Gaussian filter. Likewise, LFP signals were low-pass filtered at 1 kHz (analysis) or 150 Hz (display). For computation of power spectra and coherence, a notch filter (50 ± 1 Hz) was applied to the data. In the analysis of phase relations, the LFP was band-pass filtered in the theta (3–8 Hz) or gamma frequency range (30–90 Hz). To determine the EPSC or IPSC charge per theta cycle (Figure 5F), we detected minima of the theta component in the LFP, windows of plus or minus one-half theta period were defined according to the LFP peak of power, and current traces were integrated within these time windows.

Spectra and coherence were calculated using the density spectral power periodogram (DSPPeriodogram) function of Igor, using data segments of 1 s duration. Before analysis, data were windowed using Hanning windows with 50% segment overlap and DC value subtraction. Coherence was calculated as the cross-power spectrum of two signals, normalized by the geometric mean of the individual power spectra. Shuffling was performed by randomizing the temporal order of the LFP data points, using the linear congruential random number generator ran2 (Press et al., 2007). The significance of the differences between original data and shuffled data was evaluated by a Kruskal-Wallis test. The significance of individual coherence peaks was examined using a subsequent Wilcoxon signed-rank test with Bonferroni correction.

The autocorrelation was determined using the Correlate function of Igor and cross-checked with the Autocorrelation function of Octave. Autocorrelation (time lag range of −1 to +1 s; sampling interval of 50 μs) was computed over the total recording time (i.e., 2 min continuous recording; Figures S6C and S6D). The mean period was determined as the first peak time lag of the autocorrelogram (Figure S6D).

Phase relations were analyzed using the circular statistics tools of Igor. Phase was computed as the angular deviation between EPSC or action potential onset and theta or gamma cycle trough, using the peak of power of the LFP to determine the period. Phase locking was assumed if the distribution of angular deviations differed significantly from a circular uniform distribution (Rayleigh test).

To evaluate whether theta-gamma oscillations were nested, we performed a cross-frequency coherence (CCoh) analysis of LFP signals and synaptic currents (Colgin et al., 2009). The CCoh was computed using the Igor continuous wavelet transform procedure. A Morlet wavelet with an angular frequency ω = 6 was used. The amplitude envelope of the unfiltered LFP, IPSC and EPSC, and the phase of the unfiltered LFP were computed with the continuous wavelet transform procedure in the frequency range of 1–200 Hz.

For frequency-time representation of power plots (Figures 4B and S7B), the power was normalized by the SD at each frequency. For CCoh plots (Figures 4C and S4), the amplitude envelope was normalized by the SD at each frequency, and the phase was normalized by π.

To determine the fractional contribution of theta activity to the total power in the LFP (Figure 4B, bottom right), we calculated the proportion of experimental time in which the ratio of theta to nontheta activity was >1. All sample points fulfilling the criterion were summed, divided by the total number of sample points, and finally expressed as percentage.

Statistical significance was assessed using nonparametric tests (Wilcoxon signed-rank test for paired samples, Kruskal-Wallis test for multiple separate populations, and Rayleigh test for circular uniformity; Zar, 2010). Two-sided tests were used in all cases except in thermoinactivation experiments (in which a single-sided test was used, because a reduction of activity by cooling was expected). Differences with p < 0.05 were considered significant. Values are given as mean ± SEM. Error bars in the figures also represent SEM. Membrane potentials are given without correction for liquid junction potentials.
