# Supplementary material for: Theta-Gamma-Modulated Synaptic Currents in Hippocampal Granule Cells In Vivo Define a Mechanism for Network Oscillations
Source: Neuron. 2014 Jan 8;81(1):140–52. doi: 10.1016/j.neuron.2013.09.046 (PMC3909463; doi:10.1016/j.neuron.2013.09.046)
Supplement: Document S1. Figures S1–S7 [file mmc1.pdf]

Neuron, Volume *81*

Supplemental Information

Theta-Gamma-Modulated Synaptic Currents in Hippocampal Granule Cells In Vivo Define a Mechanism for Network Oscillations

Alejandro Javier Pernía-Andrade and Peter Jonas

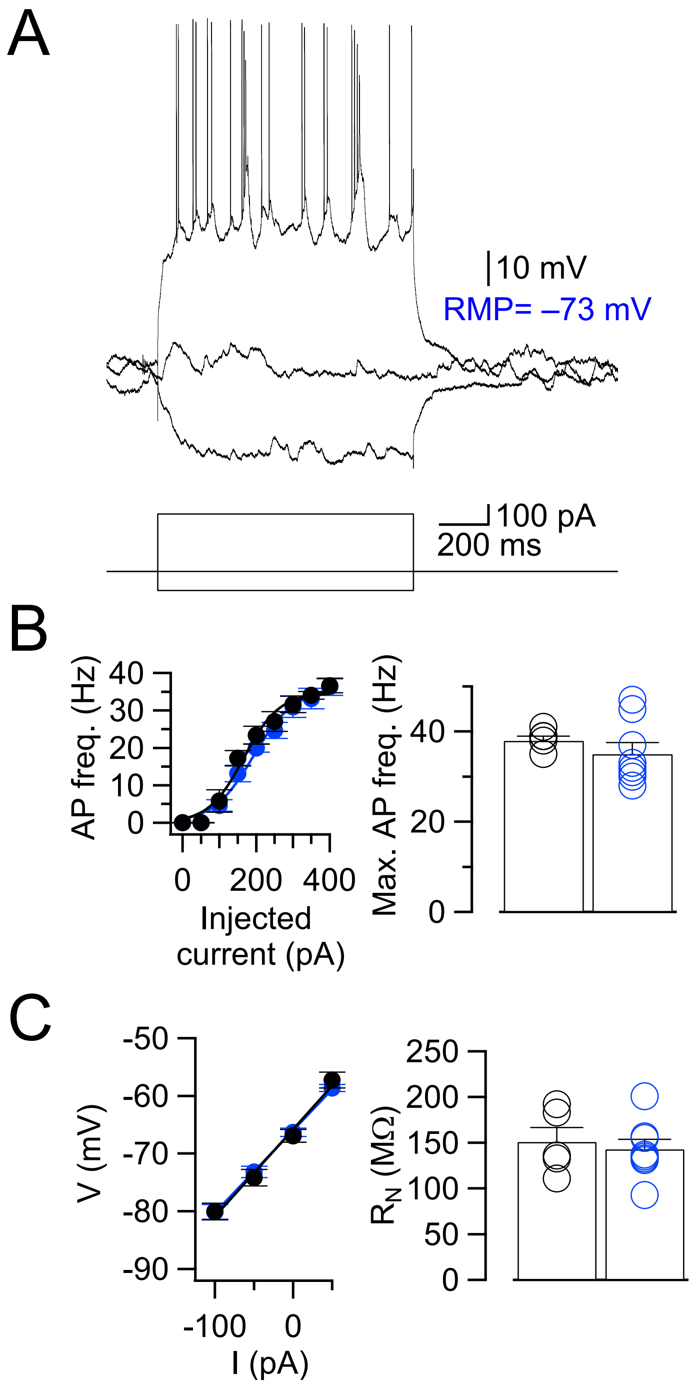

Figure S1. Action potential phenotype of GCs in vivo, related to Figure 2.
(**A**) Responses of a GC to de- and hyperpolarizing currents. Upper traces, membrane potential; lower traces, injected currents. Traces were recorded from an awake rat.

(**B**) Action potential phenotype of hippocampal GCs in vivo. Left, plot of action potential frequency against injected current (F-I curve). Right, summary bar graph of maximal action potential frequency.
(**C**) Input resistance of hippocampal GCs in vivo. Left, plot of membrane potential at the end of a 1-s pulse against injected current (V-I relation). Data points were fit by linear regression. Right, summary bar graph of input resistance, as determined from the slope of the V-I relation. Bars indicate mean ± SEM, circles represent data from individual experiments. Black, anesthetized (5 cells); blue, awake rats (8 cells).

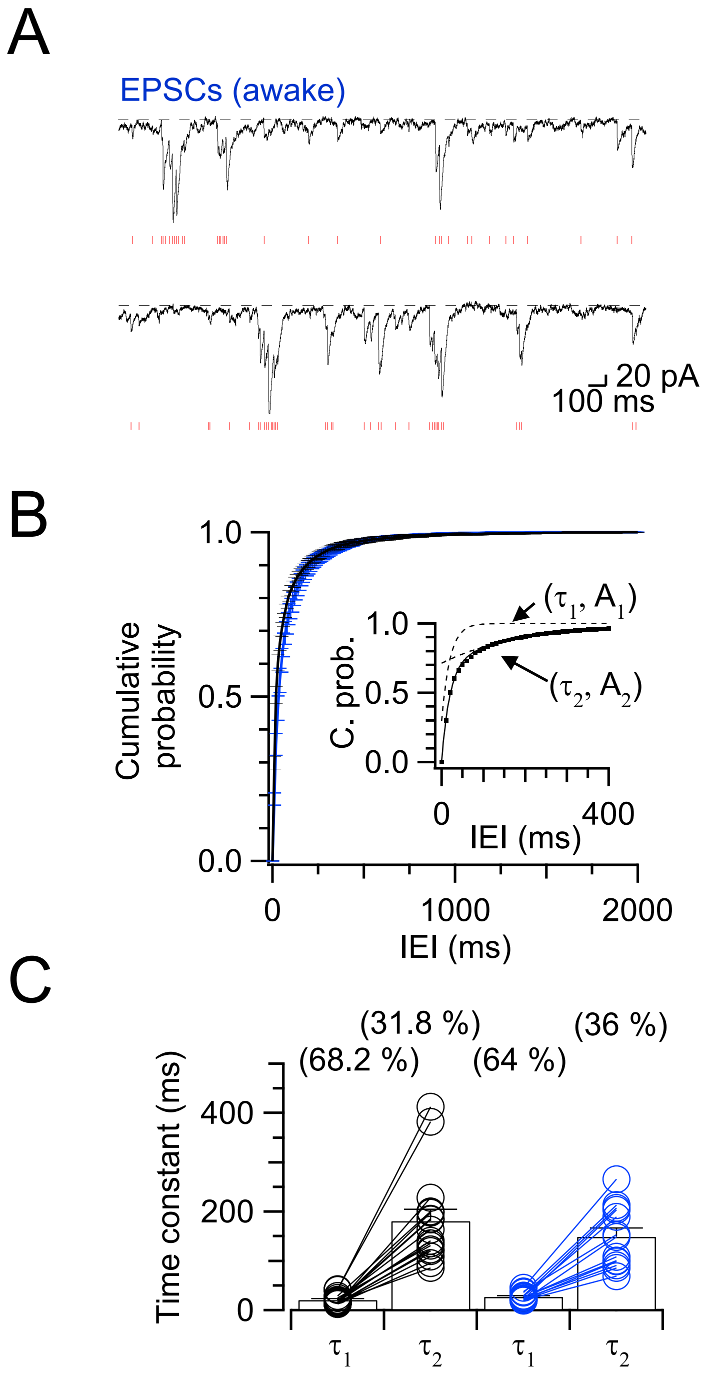

Figure S2. Bursts of EPSCs generated at theta frequency in hippocampal GCs, related to Table 1.

(**A**) Analysis of temporal structure of EPSCs by deconvolution. Two consecutive traces of EPSCs in an awake rat. Red vertical ticks indicate the onset times of detected

events. The estimated false positive rate was 0.17 points per second under these conditions.

(**B**) Cumulative probability distribution of IEI of EPSCs. Inset shows expanded view of the left portion of the distribution in a representative experiment, superimposed with fitted biexponential curve (continuous line) and individual components (dashed lines). (**C**) Time constants of fast and slow components (τ_1 and $\tau_{2,}$ respectively) of the IEI values. Numbers in parentheses indicate the corresponding amplitude contributions (A_1 and A_2, respectively). Black, anesthetized (15 cells); blue, awake rats (13 cells). Bars indicate mean ± SEM, circles represent data from individual experiments.

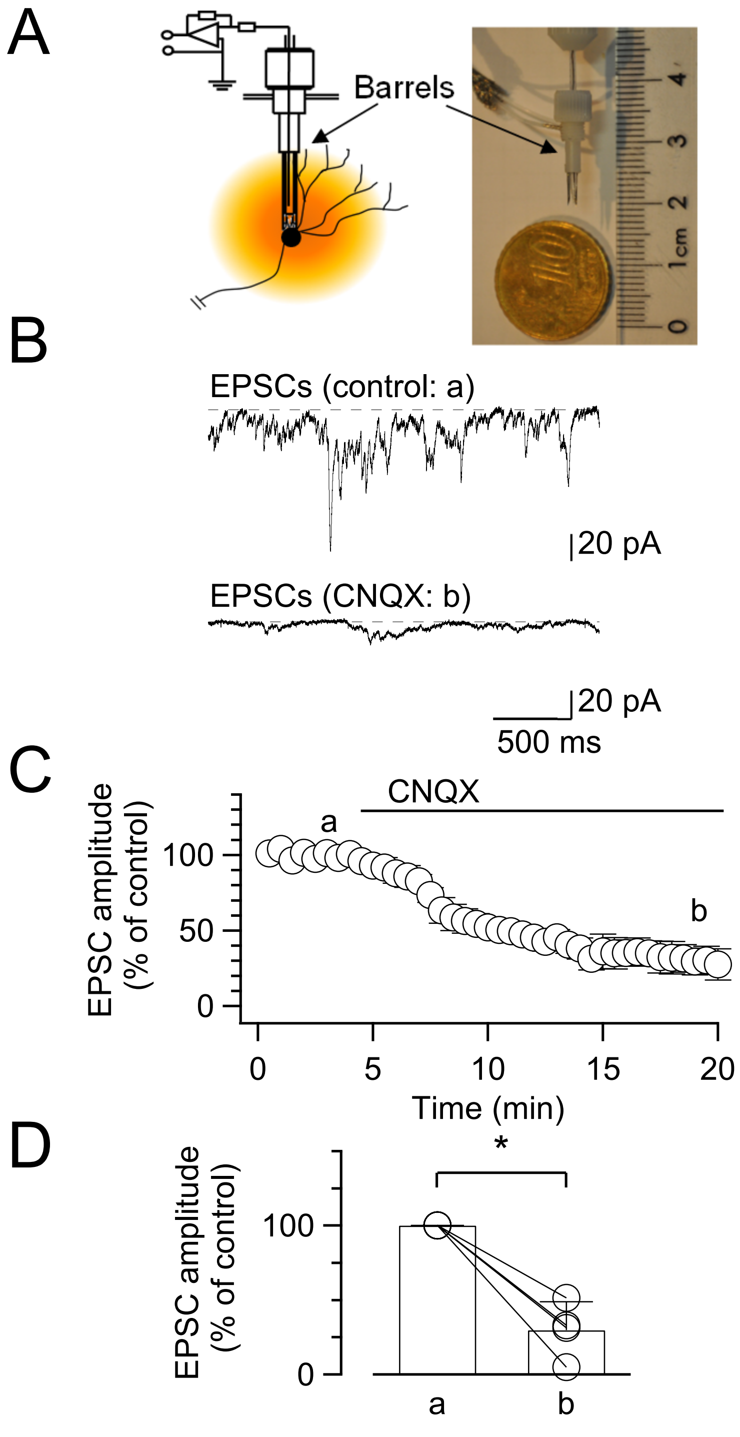

Figure S3. EPSCs in GCs in vivo are mediated by AMPARs, related to Figure 3.
(**A**) Perfusion system for antagonist application in vivo. Left, schematic illustration; right, photomicrograph of the double-barrel system.

(**B**) EPSCs at –70 mV under control conditions (top) and in the presence of 10 μM CNQX (bottom).
(**C**) Mean EPSC amplitude plotted versus time during application of 10 μM CNQX. Data from 4 experiments; symbols represent mean ± SEM; labels a and b indicate time points of traces shown in B.
(**D**) Summary bar graph illustrating the effects of CNQX. Note that CNQX blocks a large proportion of inward current in the majority of cells. Data were obtained from anesthetized rats (4 cells). * indicates $P < 0.05$. Bars indicate mean ± SEM, circles represent data from individual experiments.

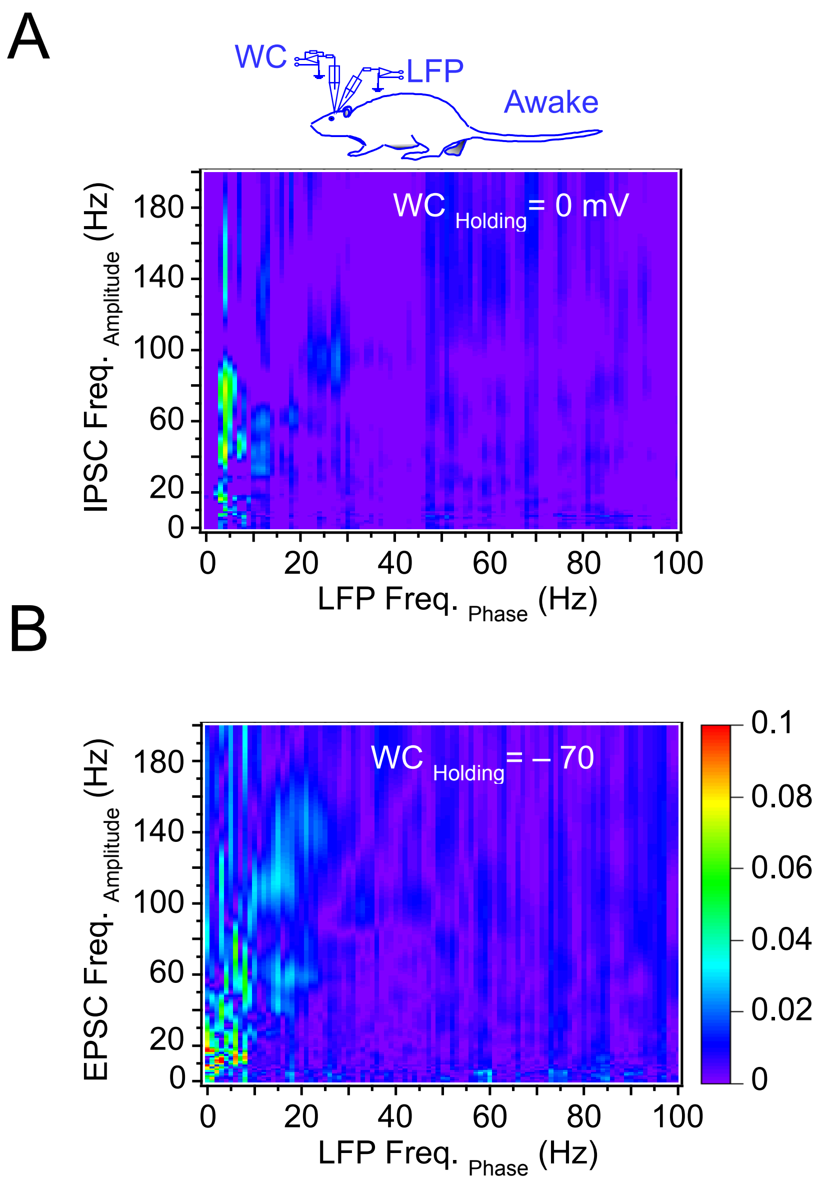

Figure S4. Evidence for nested theta–gamma oscillations in the dentate gyrus of awake rats, related to Figure 4, Figure 5, and Figure 6.

(**A**) Average cross-frequency coherence analyses between IPSC amplitude envelopes and the corresponding LFP phase. Note a prominent coupling between the IPSC amplitude envelope at 30–90 Hz and the LFP phase at 3–10 Hz and the presence of distinct bands in IPSC amplitude envelope, probably corresponding to fast and slow gamma rhythm described previously (Colgin et al., 2009).

(**B**) Average cross-frequency coherence analyses between EPSC amplitude envelopes and the corresponding LFP phase. Note the strong coupling between the EPSC amplitude envelope at 3–20 Hz and the LFP phase at 3–10 Hz.

Data from 5 cells (same experiments as shown in Figure 5). Coupling strength is color-coded (calibration bar on the right).

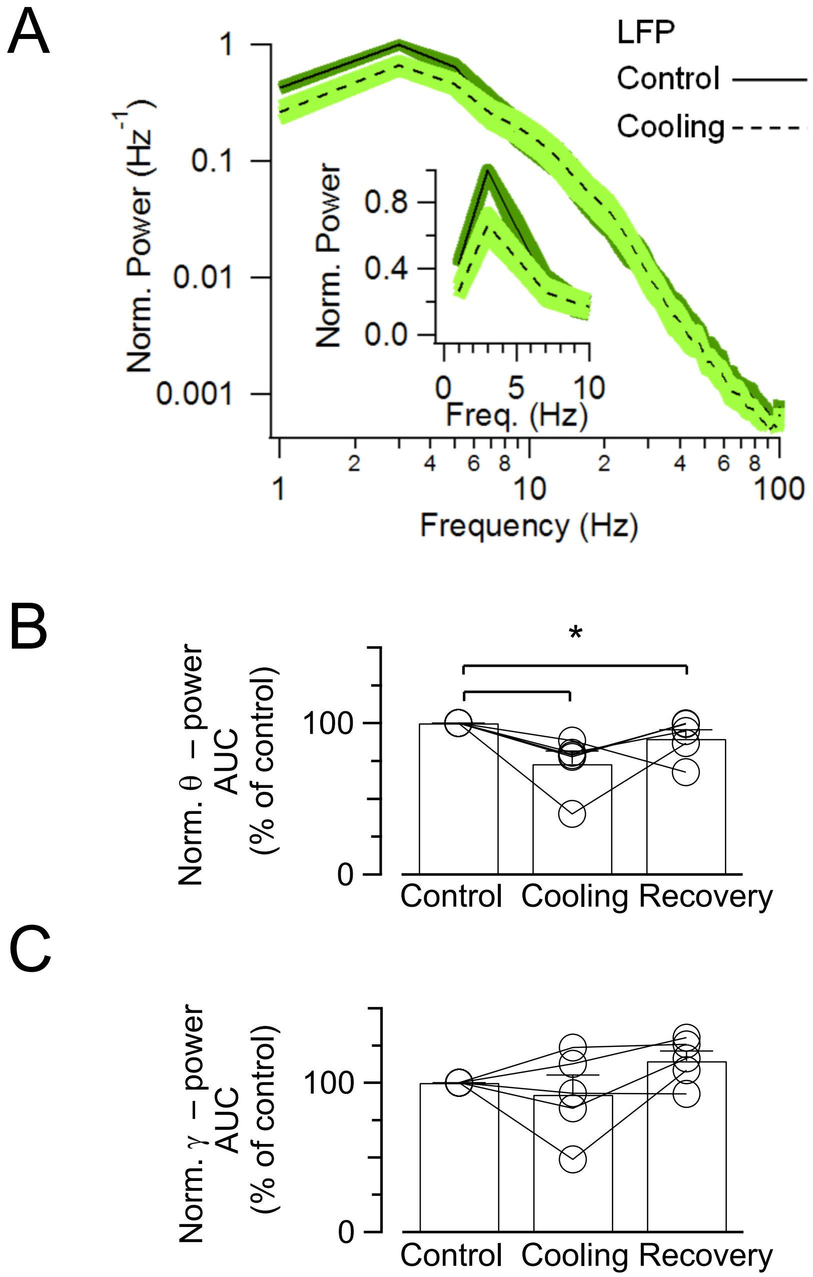

Figure S5. LFP power in the dentate gyrus is reduced by thermoinactivation of the ipsilateral entorhinal cortex, related to Figure 4.

(**A**) Average power spectrum of the LFP signal. Note that the LFP contains both theta and gamma frequency components. Continuous line, control spectrum; dashed line, spectrum during thermoinactivation. Shaded areas indicate SEM. Main graph shows

spectrum on double-logarithmic scale, inset shows same data on linear scale in the range of 0–10 Hz.
(**B**) Summary of the effects of cooling on theta power. Summary graph shows area under the curve (AUC) in the range of 3–8 Hz in control conditions, during thermoinactivation, and after recovery.
(**C**) Summary of the effects of cooling on gamma power. Summary graph shows AUC in the range of 30–90 Hz.
Both theta and gamma power were normalized to the respective control values before thermoinactivation. * indicates $P < 0.05$. Bar graphs represent mean ± SEM, circles indicate data from individual cells. Data from the same cell are connected by lines. Data were obtained from anesthetized rats (5 cells).

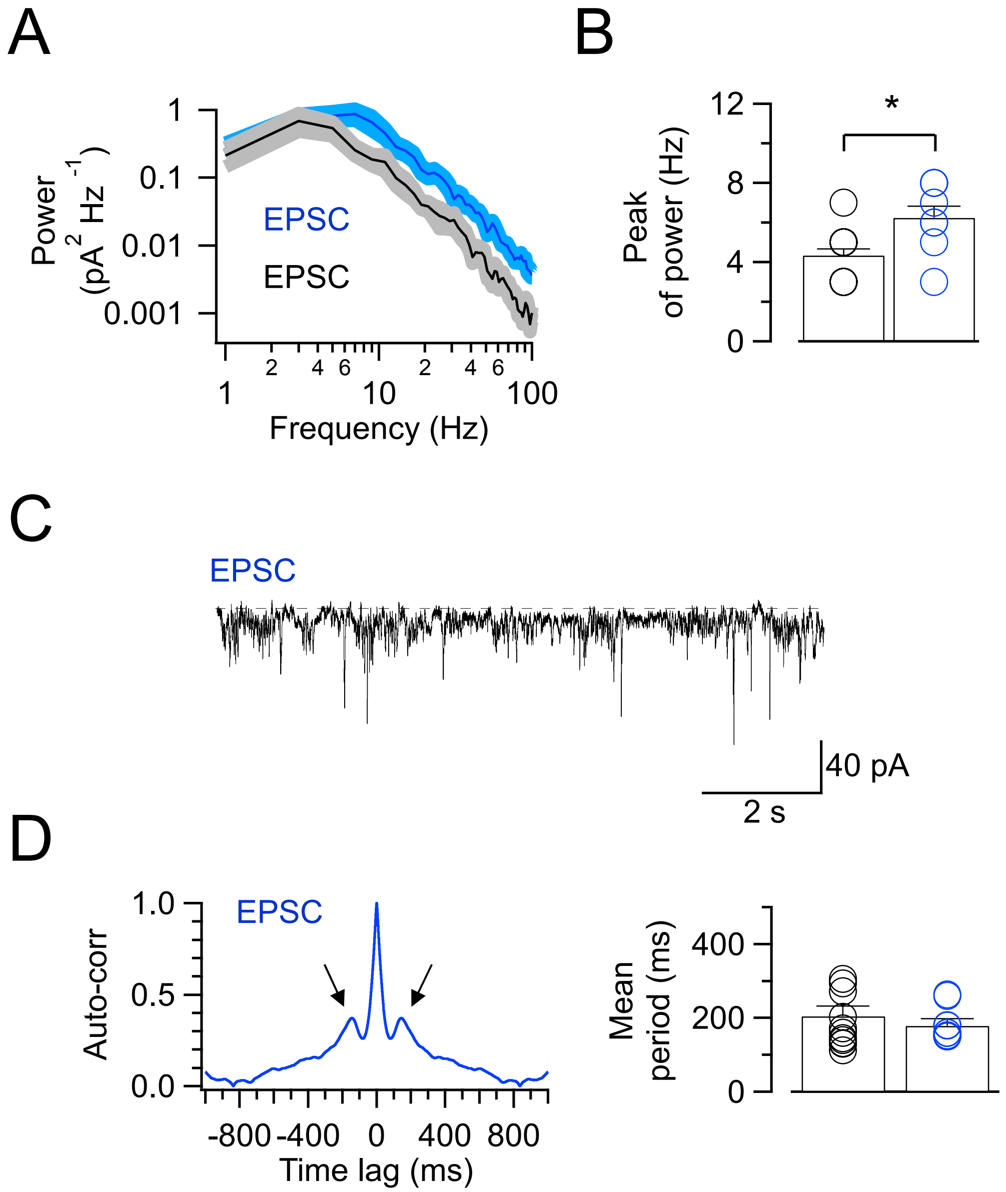

Figure S6. Power spectrum and rhythmicity of EPSCs in GCs, related to Figure 5.
(**A**) Average power spectrum of EPSCs under voltage-clamp conditions. Black, anesthetized; blue, awake animals. Shaded areas indicate the SEM.
(**B**) Summary bar graph of the frequency corresponding to maximal power. The theta peak in anesthetized animals may correspond to transient periods of high theta–delta power ratio (Klausberger et al., 2003). * indicates $P < 0.05$.
(**C**) Representative 10-s segment of a 2 min of continuous EPSC recording in a GC in awake conditions. Notice the presence of bursts of EPSCs.
(**D**) Left, rhythmicity of EPSCs as revealed by autocorrelation analysis of 2 min of continuous EPSC recording in the representative experiment shown in C. Peaks in the

autocorrelogram are indicated by arrows. Right, summary bar graph of the mean period estimated from the first peak of the autocorrelation function. Bars indicate mean ± SEM, circles represent data from individual experiments. Black, anesthetized (15 cells); blue, awake animals (13 cells).

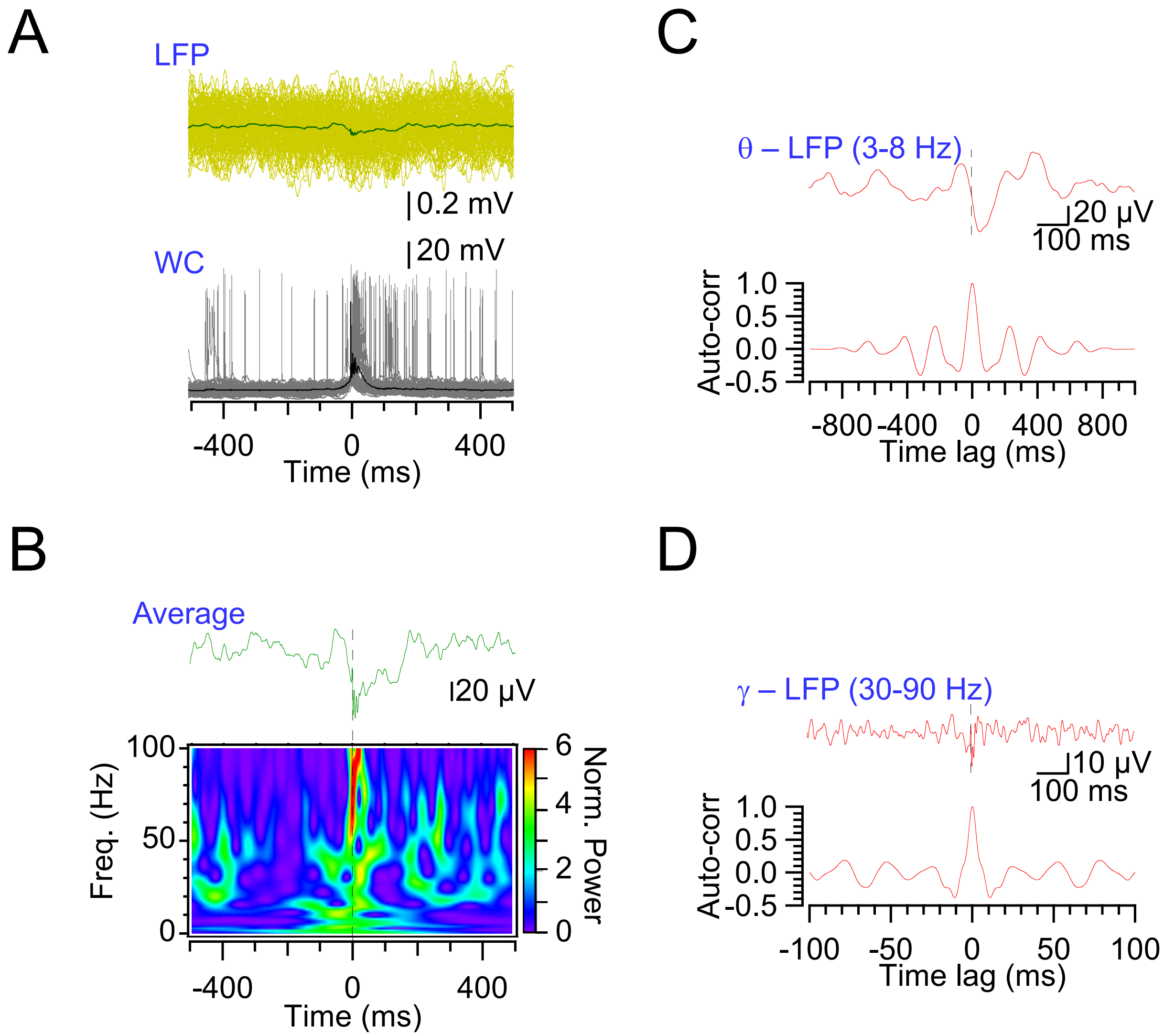

Figure S7. Spike-triggered LFP analysis reveals that action potentials are temporally coupled to theta-gamma oscillations, related to Figure 6.

(**A**) Top, action potential-triggered field potential traces (light green), superimposed with the average trace (dark green). Bottom, GC membrane potential recording with action potentials at time 0. A total of 152 action potentials (burst and single spikes) were detected in three GCs in awake rats. Both membrane potential traces in GCs and LFP traces were aligned to the action potential peak (corresponding to time 0) and averaged over a time window of ±0.5 s.

(**B**) Top, action potential-triggered LFP average trace (low-pass filtered at 150 Hz) shown at expanded voltage scale. Bottom, frequency–time representation of normalized power of the action potential-triggered LFP signal. Note the presence of both theta and gamma frequency components in the LFP at time points around the action potential.

(**C**) Analysis of theta components of action potential-triggered LFP. Top, average LFP band-pass filtered at 3–8 Hz. Bottom, autocorrelogram of the trace shown on top. Note the presence of side peaks in the autocorrelation function, indicating regularity in the data at theta frequency.

(**D**) Analysis of gamma components of action potential-triggered LFP. Top, average LFP band-pass filtered at 30–90 Hz. Bottom, autocorrelogram of the trace shown on top. Note the presence of apparent side peaks in the autocorrelation function in the gamma frequency range.
